# FortiColos – a multicentre study using bovine colostrum as a fortifier to human milk in very preterm infants: study protocol for a randomised controlled pilot trial

**DOI:** 10.1186/s13063-019-3367-7

**Published:** 2019-05-22

**Authors:** Agnethe M. Ahnfeldt, Nana Hyldig, Yanqi Li, Susanne Soendergaard Kappel, Lise Aunsholdt, Per T. Sangild, Gitte Zachariassen

**Affiliations:** 1 Section of Comparative Pediatrics and Nutrition, IVH, University of Copenhagen, Frederiksberg, Denmark; 2 Hans Christian Andersen Children’s Hospital, Odense University Hospital, Odense, Denmark; 3 Department of Neonatology, Rigshospitalet, Copenhagen, Denmark; 4 University of Southern Denmark, Odense, Denmark; 5 OPEN Odense Patient data Explorative Network, Odense University Hospital, Odense, Denmark

**Keywords:** Bovine colostrum, bovine colostrum-based fortifier, bovine milk-based fortifier, fortification of human milk, mother’s own milk, human donor milk, nutrition, growth, very preterm infants, necrotizing enterocolitis, late-onset sepsis, feeding intolerance

## Abstract

**Background:**

Very preterm infants (< 32 weeks gestation) have a relatively high nutrient requirement for growth and development. The composition of human milk is often inadequate to ensure optimal growth so it is common to fortify human milk for very preterm infants with nutrient fortifiers based on bovine milk. However, there are concerns that bovine milk-based fortifiers may increase the risk of feeding intolerance, necrotizing enterocolitis and late-onset sepsis. We hypothesize that a bovine colostrum-based product is a suitable alternative to bovine milk-based products when used as a fortifier to human milk in very preterm infants.

**Methods/Design:**

In an open-label multicentre randomised controlled pilot trial, 200 very preterm infants (26 + 0 to 30 + 6 weeks gestation at birth) will be randomly allocated to a bovine colostrum-based or a bovine milk-based fortifier added to mother’s own milk and/or human donor milk. Outcomes are growth rate, incidence of necrotizing enterocolitis and late-onset sepsis, a series of paraclinical endpoints, and practical feasibility of using the novel fortifier for very preterm infants.

**Discussion:**

The optimal enteral diet and feeding regimen for very preterm infants remain debated; this clinical trial will document the feasibility, safety and preliminary efficacy of using bovine colostrum, rich in nutrients and bioactive factors, as a novel fortifier for human milk to very preterm infants*.* Data on infant growth, metabolism, gut function and immunity will be assessed from clinical data as well as blood and stool samples.

**Trial registration:**

Registered retrospectively 25 May 2018 at ClinicalTrials.gov: NCT03537365.

**Electronic supplementary material:**

The online version of this article (10.1186/s13063-019-3367-7) contains supplementary material, which is available to authorized users.

## Background

Very preterm infants (< 32 weeks gestation) have a relatively high nutrient requirement for growth and development. Poor growth in the postnatal period is associated with later impaired neurodevelopment [1–3], metabolic disorders [4] and short stature [5]. Provision of adequate energy and nutrients (e.g. protein, minerals, vitamins) helps to prevent postnatal growth restriction [6]. Parenteral nutrition may initially be needed to support optimal nutrient intake in very preterm infants. Long-term parenteral nutrition is associated with higher risk of late-onset sepsis (LOS) [7], while a too fast increase in enteral nutrition may predispose to feeding intolerance and necrotizing enterocolitis (NEC) [8]. Further, excessive weight gain may predispose to metabolic and cardiovascular disorders later in life [9, 10]. It therefore remains a difficult task to optimize the transition to enteral nutrition, achieve adequate nutrient intake, minimise LOS and NEC, and achieve optimal growth in the postnatal period of very preterm infants [6, 7, 11].

Mother’s own milk is considered the best source of enteral nutrition for very preterm infants [12], and if mother’s own milk is not available or production is insufficient during the first weeks after preterm birth, human donor milk is recommended as the second choice. A protein intake of 3.5–4.5 g/kg/day is suggested for extremely and very preterm infants to achieve a postnatal growth rate similar to the intrauterine growth rate [13]. However, this recommended level of protein intake is seldom met by feeding on mother’s own milk and/or human donor milk alone. Furthermore, the amount of energy and protein varies widely in human milk both between mothers and throughout the lactation period [14, 15]. It has therefore become standard practice to enrich mother’s own milk and human donor milk with a nutrient fortifier to support growth, bone mineralization and neurodevelopment in very preterm infants [3, 16, 17].

Concerns have been raised that currently available fortifiers, based on bovine milk products derived from processed bovine milk and vegetable components, may increase feeding intolerance and the risk of developing NEC [18, 19]. A fortifier based on concentrated human donor milk has recently become available and very preterm infants fed exclusively human milk (mother’s own milk and/or human donor milk) fortified with the human donor milk product showed lower incidences of NEC and sepsis compared with infants fed diets partly or fully consisting of bovine-based products [20–22]. However, it remains unclear if bovine milk-based fortifiers (BMF) added to human milk (without any supplemental formula feeding) is a problem, and the high cost of the fortifier based on human milk may prevent its widespread use in the future [23]. A recent randomised control trial in preterm infants showed no difference in feeding tolerance when a human milk-based fortifier was compared with a bovine milk-based fortifier and vegetable products [24]. Fortifiers based on milk from non-bovine species are also being tested (e.g. donkey) to investigate whether bovine milk protein constitutes a specific problem for preterm infants [25].

Bovine colostrum (BC) is the first milk from cows after parturition and, like human colostrum, it contains much higher levels of protein, antimicrobial factors, immunoregulatory factors and trophic factors than mature milk (e.g. immunoglubolins, lactoferrin, lysozyme, lactoperoxidase, osteopontin, transforming growth factor, insulin-like growth factors, epidermal growth factor) [26]. These components may improve gut maturation, protection and nutrient assimilation, even across species. Used as the first feed or as a fortifier to human milk, intact BC improves gut maturation and NEC resistance in preterm pigs [27–29] and preliminary studies indicate that it is well tolerated also in preterm infants [30, 31].

On this background, we hypothesize that a powdered BC-based fortifier (BCF) for human milk can induce similar growth and better NEC and LOS resistance than a conventional, powdered BMF. A pilot trial is required to test the practical feasibility and safety of using BCF, e.g. that growth rates and clinical variables are similar to those of BMF. Further, the pilot trial is required to calculate the sample size for a later, larger randomised control trial with NEC and LOS as the primary outcomes.

## Methods/Design

### Study setting

**Fig. 1:**
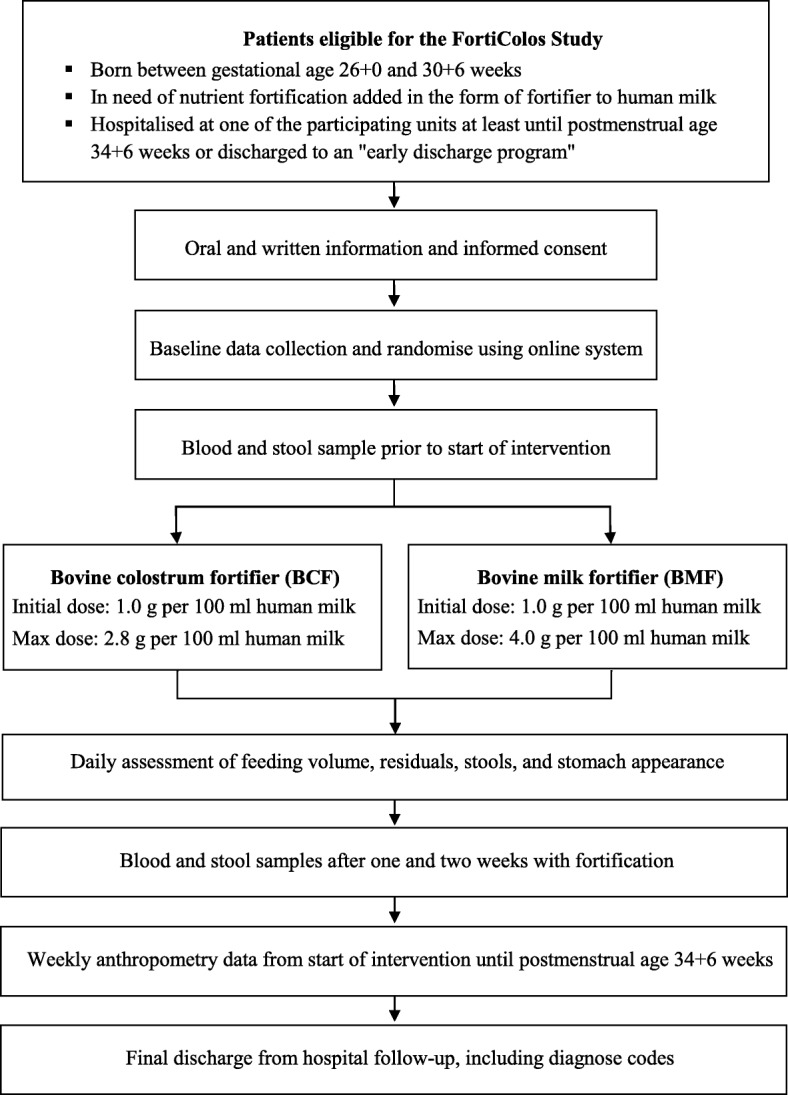
Flow of participants and data collection in the FortiColos Study

**Fig. 2:**
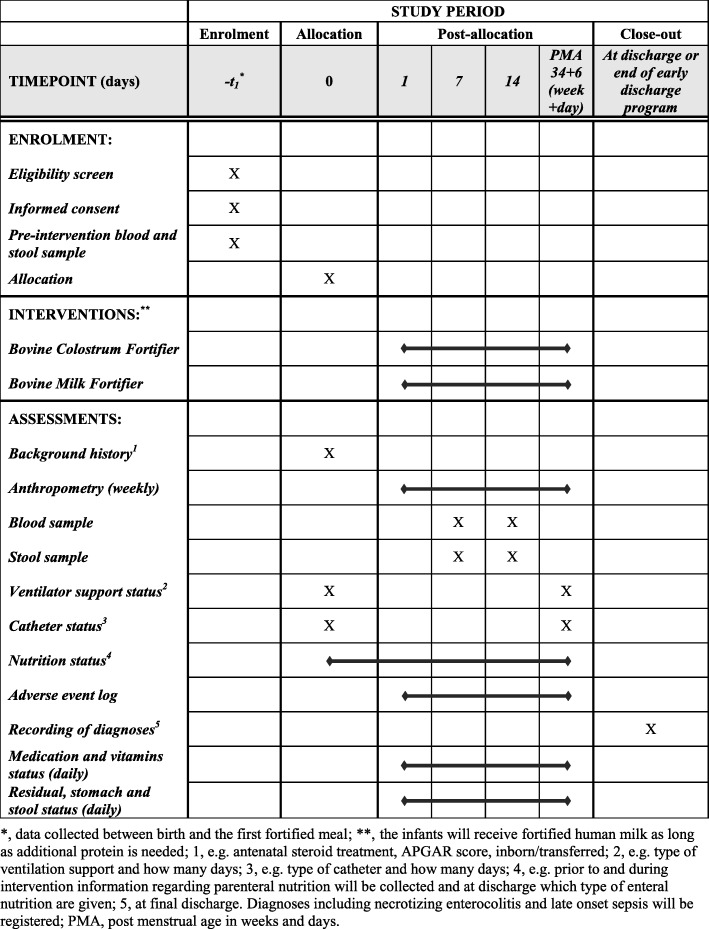
SPIRIT figure for FortiColos showing the schedule of enrolment, interventions and assessments

This study is designed as an open-label randomised controlled multicentre pilot trial of BCF compared with a conventional BMF, both used to fortify human milk, i.e. mother’s own milk and/or human donor milk (Figs. 1 and 2).

### Study population

Study participants will be recruited from multiple neonatal intensive care units and neonatal care units in Denmark. Very preterm infants born between gestational age 26 + 0 and 30 + 6 weeks will be recruited if they are in need of nutrient fortification to achieve optimal growth rates, as judged by the responsible clinical personnel. The infant should stay at one of the participating units at least until postmenstrual age (gestational age plus weeks and/or days since birth) 34 + 6 weeks, before being transferred to a non-participating unit. Infants participating in an early discharge programme (discharged home for breastfeeding establishment with a nasogastric tube) can also participate in the study until discharged. Exclusion criteria are major congenital anomalies and birth defects, gastrointestinal surgery and formula feeding prior to randomisation.

### Intervention

Introduction of enteral nutrition will follow the standard practice at the participating neonatal care unit and neonatal intensive care units. At all units, the mothers are encouraged to express breast milk as soon as possible after birth and continue expressing, so the infants will receive as much mother’s own milk as possible. Human donor milk will be used when mother’s own milk is not available or insufficient in amount. Fortification of human milk (both mother’s own milk and/or human donor milk) will start when enteral nutrition reaches a volume of at least 100 and maximum 140 mL/kg/day, unless blood urea nitrogen (BUN) values are > 5 mmol/L. In such cases, fortification may be delayed and started at higher feeding volumes. Eligible infants will be randomly assigned to one of two groups, namely human milk will be fortified with either BCF (Bovine colostrum, Biofiber, Gesten, Denmark) or a commercially available BMF (PreNAN FM85, Nestlé, Vevey, Switzerland). Table 1 shows the composition of macronutrients and bioactive components in BCF compared with the values in mature bovine milk, human colostrum, human term and preterm milk [32–39]. The control BMF fortifier is currently used for very preterm infants at all Danish neonatal units. The nutrient compositions of the two fortifiers are compared in Table 2. On the first day of intervention, 1.0 g of fortification is added to 100 mL of human milk in both groups. In the following days, the amount of BCF and BMF is upregulated with a planned speed to ensure that the same protein level is provided from both fortifiers to a maximum of 1.4 g per 100 mL of human milk, corresponding to 2.8 g of BCF and 4.0 g of BMF. The units may choose to individualise the fortification level according to the amount of protein in mother’s own milk measured on a weekly basis, or they may use standard fortification based on an assumed composition of human milk [15]. The infants will receive fortified human milk as long as additional protein is needed, according to postnatal growth rates and breastfeeding establishment. Both groups will be supplemented with phosphorus, iron, multivitamins and vitamin D according to standard recommendations.

**Table 1:** Macronutrient composition and bioactive factors in bovine and human colostrum, term and preterm milk

	Bovine colostrum	Human colostrum	Preterm human colostrum	Term bovine milk	Term human milk	Preterm human milk
Protein, g/L	60–135^a^	11–32^c^	19^f^	34^c^	9–12^g^	12.7^f^
Casein, g/L	26^b^	3.0–5.6^c^		2~30^c^	4–4.8^g^	
Whey, g/L	35–119^a^	4.3–11.1^c^		4~50^c^	6–7.2^g^	
α-Lactalbumin, g/L	2.04^c^	2.56^c^		1–1.5^c^	2–3^c^	
β-Lactoglobulin, g/L	14.3^c^	None^c^		3–4^c^	None^c^	
Lactoferrin, g/L	1.0–2.0^c^	5.0–7.0^c^		0.01–0.1^c^	1.0–2.0^c^	
Immunoglobulins, g/L	20–150^c^	1.14–20^c^		0.6–1.0^c^	1.2^c^	
Lactoperoxidase, mg/L	11–45^c^	5.17^c^		13–30^c^	5.17^c^	
Osteopontin, mg/L	Not determined^c^	1493.4^c^		18^c^	138^c^	
Lysozyme, mg/L	0.14–0.7^c^	270–430^c^		0.07–0.6^c^	160–460^c^	
Superoxide dismutase, U/mL	0.06–2.88^c^	18.7–22.5^c^		0.06–2.88^c^	11.2^c^	
Platelet-activating factor-acetylhydroxylase, μg/L	None^c^	0.95–1.19^c^		None^c^	1.16–1.21^c^	
Alkaline phosphatase, μkat/L	6.84^h^	1.79^c^		4.49^c^	0.92^c^	
Transforming growth factor-β, μg/L	150–1150^c^	1366^c^		13–71^c^	953^c^	
Insulin-like growth-I, μg/L	49–2000^c^	29–49^c^		4–150^c^	3–6^c^	
Insulin-like growth-II, μg/L	400–600^c^	10.5^c^		50–100^c^	35^c^	
Epidermal growth factor, μg/L	4–324.2^c^	35–438^c^		2–155^c^	20–111^c^	
Lactose, g/L	18.9–32^a^	44–59^d^	57–74^f^	49^a^	67–78^g^	73^f^
Fat, g/L	50–80^a^	20–29^e^	26^f^	37^a^	32–3^g^	35^f^

^a^Abd El-Fattah AM [32], ^b^Korhonen HJ [33], ^c^Chatterton DEW [34], ^d^Espinosa-Martos I [35], ^e^Jensen RG [36], ^f^Boyce C [37], ^g^Ballard O [38], ^h^Zanker IA [39]

**Table 2:** Amount of energy, protein, minerals and vitamins in PreNAN FM85 powder and the used bovine colostrum powder, as indicated when maximum fortification is reached

	Bovine colostrum Pr. 2.8 g	PreNAN FM85 Pr. 4.0 g
Energy, kcal	13	17
Protein, g	1.4	1.4
Carbohydrate, g	0.6	1.3
Fat, g	0.6	0.72
Calcium, mg	25.8	76
Phosphorus, mg	22.7	44
Zink, mg	0.20	0.96
Iron, mg	0	1.8
Vitamin D3, μg	0	3.5
Vitamin A, μg	27.8	333
Vitamin E, mg	0.05	3.8
Vitamin C, mg	0	19

### Data collection and management

We will collect data from birth until final discharge. During the intervention period, nurses will record information regarding feeding volumes, residuals (volume and colour), and appearance of stomach (any distension, colour and vessel appearance) and stools (amount, colour and texture), using a modified version of the Infant Stool Form Scale [40] and the COMFORTneo Scale [41]. Data will be collected using paper case report forms which are entered into an online database (REDCap) [42]. The electronic database is stored in a secure server at the Region of Southern Denmark.

Blood and stool samples will be collected before the first fortified meal, and at 1 and 2 weeks after start of fortification, and stored at − 60 °C to − 80 °C. All samples will subsequently be transported to a centralized biobank at the University of Copenhagen for later analysis. Randomised infants, discontinued early from the randomised type of fortification (according to clinical judgment or parental request, will with parental acceptance continue collecting data), will be considered as ‘off-study fortifier’ but ‘on study’ and followed until discharge with sampling of blood and faeces, if possible. Discontinuation due to withdrawal of parental consent or loss to follow-up will also be recorded.

### Outcome measures

#### Primary outcome

The primary clinical focus in this pilot trial is the evaluation of both body growth and incidence of NEC and LOS. The proposed primary outcomes in a later larger-scale randomised controlled trial will be NEC- and LOS-free survival, but this pilot study does not allow for a full investigation of these endpoints with adequate statistical power. Since the main reason to fortify human milk is to improve postnatal growth rates, it will also be important to document whether the novel BCF fortifier can induce infant growth rates similar to those of the conventional BMF. Body growth will be recorded as weight gain in grams from birth to discharge from hospital. Weight at different time points will be calculated into z-scores according to Niklasson and Albertsson-Wikland [43]. Delta z-scores will be used to evaluate growth and for comparison between groups. NEC is defined as Bell’s stage II or above [44]. LOS is defined as clinical signs of infection (e.g. increased heart rate, increased need of oxygen and episodes with apnoea, fluctuation of temperature and/or pale colour of the skin) with or without increased CRP after age 2 days, and antibiotic treatment for ≥ 5 days, or shorter if the infant died, with or without one positive bacterial culture in blood or cerebral spinal fluid.

#### Secondary outcomes

A series of secondary outcomes will be evaluated at different time points. Days on parenteral nutrition are defined as number of days the infant receives intravenous intakes of protein and/or lipid and/or glucose. Feeding volume when fortification is initiated, amount of fortification at each meal and duration of fortification will be registered. Time to reach full enteral nutrition (in days) is defined as the time when at least 150 mL/kg/d is reached and parenteral nutrition has been discontinued. Feeding intolerance is defined as proportion of days with a nutrition volume less than 50% of the total planned volume per day, measured during intervention. Stomach, residuals and stool appearance will be evaluated each day during intervention. Length of hospital stay is defined as days from birth until final discharge. Body length and head circumference will be measured from birth and once a week during intervention and at final discharge. BUN, blood minerals and blood haemoglobin will be collected from medical records during the intervention period. Plasma levels of amino acids, intestinal fatty acid binding protein, neutrophil extracellular trap components, lactoferrin and interleukin (IL) will be measured prior to and 1 and 2 weeks after start of fortification. Faecal composition of microbiota and faecal levels of IL-8, calprotectin (S100-A8/9) and metabolites (short-chain fatty acids) will be measured in faecal samples taken just prior to and 7 and 14 days after start of fortification.

### Randomisation and blinding

Each infant will be randomly allocated by a local member of the research team who accesses an online randomisation programme (REDCap) via a server at the Region of Southern Denmark. A computer-generated randomisation sequence will be used with a 1:1 allocation, random block sizes of 4–6, and stratified by small-for-gestational-age (SGA, yes/no), where SGA is defined as a birth weight z-score less than two standard deviations [44]. The random allocation sequence will be generated by an external data manager not involved in the trial. In case of multiple births, all siblings will be allocated to the same group, randomised by the first-born infant. Blinding is not possible in this trial, and the two powdered fortifiers can be distinguished by colour and texture. The schedule of enrolment, intervention and data collection are described in Figs. 1 and 2.

### Statistical analysis

#### Sample size

The sample size in this pilot trial is set at *n* = 100 for each group (total 200) based on pragmatic evaluations for comparison between groups on infant growth, laboratory outcomes (clinical and paraclinical) and the study feasibility outcomes. If the primary endpoint had been NEC- and LOS-free survival, the total sample size should have been 1498 participants according to a power calculation, based on an aim to test a 50% reduction in NEC incidence and a 25% reduction in LOS incidence at discharge with 80% power and a two-sided 0.05 level of significance, and assuming a NEC incidence of 6% and a LOS incidence of 30%.

#### Analysis

Statistical analyses will be performed as both intention-to-treat and per protocol analyses. Per protocol analysis will include infants treated with the randomised type of fortifier for a minimum time of 2 weeks. Continuous outcomes will be summarized as mean and standard deviation (e.g. body weight) or median and interquartile range (e.g. time to reach full enteral nutrition). Binary outcomes (e.g. incidence of NEC) will be presented as counts and percentages.

To make a preliminary test of the effects of the intervention with BCF, clinical and paraclinical outcomes will be compared between the two groups. The estimates will be presented as relative risk and absolute risk difference, difference between means or hazard ratio, depending on the type of outcome. The estimates will be presented with a 95% confidence interval. Regression models, logistic regression for binary outcomes and Cox regression for time to event outcomes will be used to evaluate the outcomes of interest between the two groups, adjusting for SGA (stratification variable) and covariates that are known to be prognostic in relation to the outcome variable (e.g. gestational age, birth weight and sex). A Fine–Gray regression, taking competing risk of infant death into account, will be carried out as a sensitivity analysis for time to event outcomes. If relevant, sensitivity analyses will be conducted using multiple imputations to explore the potential impact of missing data. Results from all these analyses will be considered preliminary since this a pilot-scale trial. The feasibility of the study will be presented as categorical variables with three levels as displayed in Table 3.

**Table 3:** Pre-defined criteria used to decide whether, or how, to proceed with a later, larger randomised control trial. Green colour: Fully acceptable and feasible. Yellow colour: Feasibility concern, changes to be decided. Red colour: Serious feasibility concerns, clear actions must be taken before a greater randomised control trial is planned

			
Consent rate	> 70%	50–70%	< 50%
Recruitment rate	> 50%	20–50%	< 20%
Proportion of incomplete datasets	< 20%	20–50%	> 50%

### Ethics approval and consent to participate

The trial (version 2 of the protocol) has been approved by the Regional Scientific Ethical Committee of Southern Denmark on November 28, 2017 (S-20130010) and the Danish Data Protection Agency on October 4, 2017 (2008-58-0035), and registered at ClinicalTrials.gov (NCT03537365).

Parents will be given oral and written information about the trial, including its purpose, risk and benefits. They will be given sufficient time to consider participation in the trial and to have their questions answered. Written informed consent must be provided before implementation of study procedures (e.g. randomisation and blood test). The parents may withdraw their infant(s) from the trial for any reason at any time. Similarly, investigators may withdraw one or more infants, according to clinical judgments and safety assessment, or if the parents are unwilling to comply with required study procedures, or if the trial is terminated early for any reason.

### Data monitoring

#### Adverse event reporting

Very preterm infants are often seriously ill and both adverse and serious adverse events may occur during hospitalisation. All unexpected adverse events (related or unrelated to intervention) will be recorded in the case report form, including information on type of event, date of onset, end date, intensity (mild, moderate, severe), severity (yes/no) and their presumed relation to the intervention (yes/no). Serious adverse events and all suspected unexpected serious adverse reactions (SUSARs), suspected to relate to the BCF intervention or the BMF control group will be reported to the coordinating investigator and the sponsor within 24 h after the local principal investigator becomes aware of the event. The coordinating investigator is responsible for informing the Scientific Ethical Committee and the Data Safety Monitor Board of any SUSARs that occur during the trial.

#### Data Safety Monitor Board

An independent Data Safety Monitor Board (DSMB) is established to assess the trial progress and to ensure independent evaluation of possible harm to participants. The DSMB consists of three members who are respective experts in neonatology, biostatistics and methodology in clinical trials. Three pre-planned DSMB meetings are held during the recruitment period. Unscheduled meetings will be held if the coordinating investigator or the sponsor consider it necessary, e.g. if an unexpected high number of NEC or SUSARs, possibly related to the intervention, are reported. The primary role of the DSMB is to provide recommendations regarding trial modification, continuation or termination.

## Discussion

Human milk is well documented to provide the best protection against feeding intolerance, NEC and LOS [45], yet it does not contain enough nutrients to support growth according to current guidelines. Therefore, nutrient fortification of mother’s own milk and/or human donor milk has become common practice in neonatal units around the world [46]. However, it remains unclear when, how and with what product human milk should be fortified. Fortification can be done using widely different strategies [45]. Fortification is typically initiated when human milk intake reaches 100–140 mL/kg/day, with consideration of infant age and maturity. Some units practice early start of fortification, at low volumes of enteral feed (from 50 mL/kg/day), while other units do not start until later and at higher feeding volumes (e.g. at 150–160 mL/kg/d), often related to a fear of feeding intolerance and NEC. Both standard fortification (i.e. same amount of protein given to all infants) and individualised fortification (i.e. fortification adjusted to protein contents in human milk for each infant) are practiced. Further, some units use BUN values to start and adjust fortification (e.g. 3.2–5.0 mmol/L as target values [47, 48]), some start with full-strength fortification, while others start with half-strength fortification and increase to full-strength within some days [49]. Therefore, a better understanding of the optimal way to fortify human milk for very preterm infants is needed.

In this study, we chose to start fortification with BCF or BMF at a feeding volume of 100 mL/kg/day and no later than 140 mL/kg/day, if possible according the BUN values. Together with the current international guidelines for nutrient fortification, these criteria were chosen to make the procedures similar among the participating units. In Denmark, an enteral feeding volume of 150–160 mL/kg/day is generally reached for very preterm infants 7–12 days after birth (unpublished data). A specific age (number of days since birth) at initiation of fortification was not a criterion in this trial because we believe that the actual achieved feeding volume is a better marker of adequate gut maturity than infant age.

Included infants are born at different gestational ages (26 + 0 to 30 + 6 weeks) and it is well known that the smallest and most immature infants are the most challenging to feed due to feeding intolerance and co-morbidities increasing the risk of postnatal growth failure. Therefore, they would presumably also be the infants to benefit the most from a milk diet that may be more protective for the immature gastrointestinal tract. For safety reasons, we decided to start fortification only when BUN values are below 5 mmol/L. However, it remains unclear if a moderately elevated BUN value has any relation to common complications such as kidney immaturity, metabolic dysfunctions or protein overload [47]. Further studies are required to understand the predictive value of BUN measurements to guide nutrient fortification for very preterm infants.

The concept of feeding intolerance is a key issue when discussing different strategies to fortify human milk. Feeding intolerance is not a well-defined clinical parameter and a variety of methods are used to help assess if the infant appears intolerant to enteral feeding. Several studies indicate that the volume and colour of gastric residuals is a poor indicator of feeding intolerance and early signs of NEC [48, 50]. In this pilot trial, we will also evaluate possible signs of feeding intolerance by registering both the colour and volume of gastric residuals and number of days to 50% and to full enteral feeding. Feeding intolerance might be due to increased osmolarity when adding fortifiers to human milk above the accepted upper limit (< 400 mOsm/L) [51], although the evidence in support of this upper limit is weak. Prior to our study, we investigated the osmolarity in banked human donor milk (from Hvidovre Hospital, Hvidovre, Denmark) before fortification (295 mOsm/L) and after fortification with either BCF (28 g/L, 334 mOsm/L) or BMF (40 g/L, 409 mOsm/L). These values did not increase during 24 h storage at 4 °C; hence, high feed osmolarity is unlikely to be a problem in any of the groups in our study.

The secondary and paraclinical endpoints in this pilot trial aimed to support the preliminary observations regarding the clinical effects of the two different fortifiers on gut metabolism, function and immunity. Plasma amino acid levels are relevant to record because certain amino acids can be used to indicate protein overload (e.g. tyrosine) while others may reflect improved gut function and immunity (e.g. arginine, citrulline) [28–31]. Plasma levels of neutrophil extracellular trap components, lactoferrin and ILs, especially IL-6 and IL-8, are used to assess systemic immunity and inflammation, and thereby sepsis resistance [52]. Faecal composition and diversity of microbiota and of IL-8, calprotectin (S100-A8/9) and bacterial metabolites (short-chain fatty acids) are measured to reflect how each of the fortification products affect the local gut environment, bacterial metabolism and immunity.

Collectively, our protocol provides both clinical and paraclinical endpoints to test the feasibility, safety and preliminary efficacy of a novel nutrient fortifier for very preterm infants.

## Study status

Recruitment is ongoing at eight neonatal departments in Denmark. The first infant was recruited on December 14, 2017, and we expect to complete recruitment within 2 years. Please see SPIRIT Checklist for FortiColos: Recommended items to address in a clinical trial protocol and related documents (Additional file 1).

## Additional file

Additional file 1: SPIRIT Checklist for FortiColos: Recommended items to address in a clinical trial protocol and related documents. (DOCX 58 kb)

## Abbreviations

BC: bovine colostrum
BCF: bovine colostrum-based fortifier
BMF: bovine milk-based fortifier
BUN: blood urea nitrogen
DSMB: Data Safety Monitoring Board
IL: interleukins
LOS: late on-set sepsis
NEC: necrotizing enterocolitis
SGA: small for gestational age
SUSARs: severe unexpected suspected adverse reactions
